# Change in the Single Amino Acid Site 83 in Rabies Virus Glycoprotein Enhances the BBB Permeability and Reduces Viral Pathogenicity

**DOI:** 10.3389/fcell.2020.632957

**Published:** 2021-02-09

**Authors:** Chunfu Li, Yongzhi Wang, Huiting Liu, Xinghua Zhang, Dalai Baolige, Shihua Zhao, Wei Hu, Yang Yang

**Affiliations:** ^1^The State Key Laboratory of Reproductive Regulation and Breeding of Grassland Livestock, School of Life Sciences, Inner Mongolia University, Hohhot, China; ^2^Veterinary Research Institution, Inner Mongolia Academy of Agricultural and Animal Husbandry Sciences, Hohhot, China

**Keywords:** rabies virus, glycoprotein, pathogenicity, blood-brain barrier, MMPs

## Abstract

Lab-attenuated rabies virus (RABV) is a highly cellular adaptation and less pathogenic than wild-type RABV. However, the molecular mechanisms that regulate the cellular adaptation and pathogenicity remain obscure. In this work, we isolated a wild-type RABV (CNIM1701) from a rabid bovine in northern China. The original CNIM1701 was lethal in adult mice and restricted replication in cell cultures. After 20 serial passages in the brains of suckling mice, the virus was renamed CNIM1701-P20, which was safe in adult mice and replicated well in cell cultures. In addition, sequence comparison analysis of the original CNIM1701 and CNIM1701-P20 identified 2 amino acid substitutions on G protein (Lys83 → Arg83 and Pro367 → Ser 367) related to pathogenesis and cellular adaptation. Using site-directed mutagenesis to exchange Lys83 with Arg83 and Pro367 with Ser 367 in the G protein of the RABV SAD strain, the pathogenicity of rSAD-K83R was significantly decreased. Our data indicate that the decreased pathogenicity of rSAD-K83R is due to increasing the expression of RABV-G, which also induced a higher level of apoptosis in infected cells. Furthermore, the K83 mutation induced high expression of MMP-2 and MMP-9 on DCs and promoted blood–brain barrier (BBB) permeability. These results demonstrate that the pathogenesis of RABV is partially dependent on G expression and BBB permeability, which may help in the design and development of highly safe, live-RABV vaccines.

## Introduction

Rabies virus (RABV) is a non-segmented, negative-stranded RNA virus of the Rhabdoviridae, which causes rabies—a global public health threat and fatal viral zoonosis. RABVs spread to humans via biting or scratching of RABV-infected terrestrial mammals, such as bats (Ribeiro et al., 2018), dogs (Shwiff et al., 2018), and skunks (Leslie et al., 2006). As with various other infectious diseases, immunization methods for the human rabies vaccine are postexposure prophylaxis (PEP) rather than preexposure (Hampson et al., 2008). Furthermore, the appropriate use of the rabies vaccine and human rabies immune globulin (HRIG) are shown to be highly effective in preventing infections and death (Chao et al., 2017) although, due to the limitation of effective control in animal reservoirs and high vaccine costs in developing countries, more than 59,000 people still die from rabies each year (2005). According to a report, more than 95% of human deaths are reported in developing countries in Asia and Africa (Hampson et al., 2015). Although the rabies vaccine was developed in 1885, it is still a costly reagent (Rappuoli, 2014). In recent decades, modern cell culture techniques have increased the quality of vaccines, including purity, efficiency, safety, high production, and economical to produce. In view of these, a highly cell-adapted and stable RABV vaccine strain remains to be explored, and the specific nucleotide sites that promote cellular adaptation need to be further investigated.

The first licensed cell culture human RABV vaccine was SAD [a rabies virus was isolated in a rabid dog from Chao et al. (2017) Alabama in 1935], and it was grown in primary hamster kidney cells in the 1960s (Kissling, 1958; Fenje, 1960). To explore an experimental rabies vaccine, lots of street and fixed RABVs were produced to adapt to cells by this similar procedure, including Flury, CTN, and Pitman–Moore (PM). The Flury RABV low-egg-passage (LEP) and high-egg-passage (HEP) strains were established after serial passage in the chicken brain, 180 passages in chicken embryos and BHK-21 cells or chicken embryo fibroblast (CEF) cells using a RABV isolated from a girl named Flury, who died from rabies in Georgia (Koprowski et al., 1954; Barth et al., 1984). The CTN-1 was isolated from the brain tissue of a patient, and CTNCEC25 was CTN-1 well-adapted to chicken embryo cells (CECs) after 57 passages (Guo et al., 2014). The PM was adapted to human diploid cells after 52 passages and serially propagated in BHK-21 cells using the original Louis Pasteur virus (PV) passaged in rabbit brains in the United States (Wiktor et al., 1964). Besides this, the PV and challenge virus standard (CVS), which is also a subtype of the original PV, are well-adapted to cell cultures (Sacramento et al., 1992).

To date, despite more than a century of rabies vaccine development, the global major live-attenuated RABV vaccines include SAD variants (Maki et al., 2017), and the inactivated RABV vaccines involve Flury, PM, PV, and CTN (Singh et al., 2017). Previous studies prove that the pathogenicity of RABV is inversely correlated with its replication rate in tissue culture and cells (Pulmanausahakul et al., 2008). In this regard, most of the present studies explore the genetic sites that determine the cell-adapted amino acid (AA) mutations of RABV using those classical attenuated RABV strains. However, following different passages and adaptation after decades, each of these RABV vaccine strains developed dozens of subtypes, and the original wild-type RABV was omitted. Furthermore, the genetic relationship between the original virus and its derivatives is difficult to clarify today, and the initial AA mutations from the original wild-type RABV adapted to cells are hard to define.

In this study, a wild-type RABV, CNIM1701, was isolated from a rabid bovine in northern China. The original CNIM1701 was lethal in mice and, after 20 serial passages in suckling mouse brain, was renamed CNIM1701-P20. The pathogenicity of CIIM1701-P20 was significantly decreased. Genetic analysis of the complete genome of original CNIM1701, CNIM170-P20, and other rabies indicated 2 mutation sites at positions G83 and G367 in the RABV G protein. Using site-directed mutagenesis to exchange Lys83 with Arg83 and Pro367 with Ser 367 in the G protein of the SAD strain of RABV, we show here that K83R further decreased the pathogenicity of SAD L16. Furthermore, the decrement of pathogenicity was dependent on the expression level of MMPs and blood–brain barrier (BBB) permeability. These results demonstrate that the Lys83 with Arg83 mutation decreased the pathogenicity of RABV, and the permeability of the BBB is essential to protect against RABV infection.

## Materials and Methods

### Animals and Cells

C57BL/6 mice were purchased from Charles River (Beijing Vital River Laboratory Animal Technology Co., Ltd.). Experimental infectious studies were performed in strict accordance with the Guide for the Care and Use of Laboratory Animal Monitoring Committee of Hubei Province, China, and the Scientific Ethics Committee approved the protocol of Huazhong Agricultural University (protocol no. Hzaumo-2015-018). All efforts were made to minimize the suffering of the animals. Mouse neuroblastoma (NA) cells were maintained in RPMI-1640 medium (Gibco, China) supplemented with 10% fetal bovine serum (FBS) (Gibco, Australia) and 1% penicillin-streptomycin (Gibco, USA). BSR cells were maintained in Dulbecco's modified Eagle's medium (DMEM) (Gibco, China) containing 10% FBS and 1% penicillin-streptomycin. bEend.3 cells were purchased from the National Collection of Authenticated Cell Cultures. bEend.3 cells were cultured in DMEM supplemented with 10% FBS and 1% penicillin-streptomycin in a water-saturated atmosphere of 5% CO_2_ at 37°C. Cells were passaged every 3 days.

### Virus Isolation and Sequence

The RABV strains CNIM1701 and CNIM1702 were obtained from two different rabid cattle, and the CNIM1703 was isolated from a rabid sheep in the Inner Mongolia province of China. Two grams of animal brain was homogenized with 2 ml of PBS and centrifuged at 2,000 g in 4°C for 5 min. The supernatant was frozen at −80°C for virus stock, and precipitation was lysed with RNAiso plus (Takara, Japan). Total RNA was reverse transcribed into cDNA using the PrimeScript™ 1st Strand cDNA Synthesis Kit (Takara, Dalian, China). All 10 pairs of oligonucleotide primers to amplify the RABV genome are described in Table 1. The PCR products were purified by the GeneJET Gel Extraction Kit (Thermo Fisher Scientific, Lithuania) and then cloned into the pMD19T vector (Takara, Dalian, China). All the plasmids were sequenced by Huada Gene Technology Co. Ltd. (Beijing, China). The genomic sequences were assembled using DNA star5.0. Homology searches and comparisons of all the sequences were performed using the NCBI-blast. The sequences were submitted to GenBank with the accession numbers KY649620, MF172976, and HM267792.

**Table 1:** Primers used in the study.

**Primer**	**Sequence (5^′^−3^′^)^‡^**	**Positions**
RABV primer 1-F RABV primer 1-R	ACGCTTAACAACAAAATCA	1–19
	ATCTCTTCCTCAAAGTTCTT	876–857
RABV primer 2-F RABV primer 2-R	GGGTTCATAAAGCAGATAAATC	800–821
	TTATACAAGAATATCCCTGA	2,193–2,174
RABV primer 3-F RABV primer 3-R	GCTCAAACTGCCTCTGGT	2,038–2,055
	GCACCATAACATGTTTTTG	3,215–3,198
RABV primer 4-F RABV primer 4-R	TCACTTGTTTACCTCTGGA	3,128–3,146
	TATGGTATATGCCTTTCCA	4,326–4,208
RABV primer 5-F RABV primer 5-R	GGACTTAGACTTATGGACGGAA	4,068–4,089
	TAGATGACCCAGCCCTTTATAA	5,339–5,318
RABV primer 6-F RABV primer 6-R	TCCCATGAAGGACATAAGCAA	5,252–5,272
	GTTGACTGACCTTGTCTTTTAT	6,421–6,400
RABV primer 7-F RABV primer 7-R	GGAACTATACACTTATGCTGAA	6,128–6,149
	GTCTGATCTGTCTGAATAATAG	7,411–7,390
RABV primer 8-F RABV primer 8-R	ACTGGGCAAGGGCTTTT	7,214–7,230
	TCAGAAGGGTGAGGAAC	8,773–8,757
RABV primer 9-F RABV primer 9-R	CGGATGACCCAGACACCTC	8,644–8,662
	GTTGCCCACTGAACCACTCTC	10,448–10,428
RABV primer 10-F RABV primer 10-R	AGGTGGGTGGATCAAGAAGTG	10,220–10,240
	ACGCTTAACAAATAAACAA	11,928–11,910
N-qRT-PCR -F N-qRT-PCR -R	GGAAAAGGGACATTTGAAAGAA	1,121–1,142
	AGTCCTCGTCATCAGAGTTGAC	1,192–1,175
K83R-F	CGTTCA**g**GAAGAAAGCATTTCCGCC	3,615–3,638
K83R-R	GGGCGGAAATGCTTTCTT**c**TGAACG	3,639–3,615
P367S-F	ATATTAGGA**t**CTGACGGCAATGTCTTAA	4,463–4,490
P367S-R	GGATTAAGACATTGCCGTCAG**a**TCCTAA	4,493–4,466
RABV-G-F	AACTGCAGGAAAGATGGTTCCTCAGGCTCTCC	3,310–3,335
RABV-G-R	CTAGCTAGCAAATCGCCAAATCGTACGGCA	4,972–4,943
MMP-2- qRT-PCR -F MMP-2- qRT-PCR -R MMP-9- qRT-PCR -F MMP-9- qRT-PCR -R Occludin- qRT-PCR -F Occludin- qRT-PCR -R ZO-1- qRT-PCR -F ZO-1- qRT-PCR -R GAPDH- qRT-PCR -F GAPDH- qRT-PCR -R	GCAGCCCATGAGTTCGGCCAT AGCATCAGGGGAGGGCCCATA GGAGACGGCAAACCCTGCGT TGACGTCGGCTCGAGTAGGACA TCAGGTGAATTGGTCACCGAG GATAAACCAATCTGCTGCGTC TGCCATTACACGGTCCTCTG TCTGCTTTCTGTTGAGAGGCT GACAGTCAGCCGCATCTTCT GCGCCCAATACGACCAAATC	1,529–1,549 1,681–1,661 850–869 1,009–988 1,733–1,753 1,890–1,870 3,105–3,124 3,260–3,240 16–35 211–192

‡ *Underlined are restriction sites. Substituted bases are bold and written in lowercase*.

### Bioinformatic and Phylogenetic Analysis

All the currently available whole genomic RABV sequences were retrieved from the Genbank. The G gene sequence and complete genome nucleoside and AA sequences of the 59 RABVs were aligned using ClustalW in MEGA 7, and a phylogenetic tree based on the full-length genomic sequences was constructed using a distance-based neighbor joining (NJ) method with MEGA 7 (Kumar et al., 2016).

### Quantification of Viral Genomic RNA by qRT-PCR

RNA in mice and cells was used to quantify the viral genomic RNA with the Applied Biosystems® 7500 Real-Time PCR Systems (Thermo Fisher Scientific, USA) as described previously (Yang et al., 2015). In brief, total RNA from mouse brain and cells was extracted with the RNAiso Plus reagent (Takara, Dalian, China), and cDNA was synthesized by the PrimeScript™ RT reagent Kit (Takara, Dalian, China) following the manufacturer's instructions using rabies gene–specific primers (Table 1). Next, the real-time PCR assay was performed in a 20-μl reaction volume using the SYBR Fast qPCR Mix (Takara, Dalian, China), and each reaction was carried out in triplicate with approximately 100 ng of DNase-treated RNA and 5 nM of each primer pair. For counting the viral genomic RNA copies, a standard curve was generated from 10-time serially diluted plasmids expressing RABV N, and the copy numbers of viral RNA were normalized to 1 mg of total RNA.

### Virus Inoculation and Infection

Bovine and mouse brains were homogenized in phosphate-buffered saline (PBS) at a concentration of 10% (weight/volume percentage, w/v%). For virus amplification, the 1-day-old C57BL/6 mice were injected intracerebrally (IC) with 10 μl of indicated RABVs. For pathogenicity analysis, 3- or 6-week-old C57BL/6 mice were injected intramuscularly (IM) with 50 μl of indicated RABVs. Next, 3- or 6-week-old C57BL/6 mice were given the indicated RABV in the oral cavity via a needleless syringe. All the inoculated mice were monitored for 21 days for rabies-related clinical signs and euthanized, and brain samples were collected. BSR or NA cells were harvested in 12-well plates until the monolayer of cells reached 80% confluence, then infected with 1 gene copy of RABVs per cell in DMEM with 1% FBS. At 4 days postinfection, the BSR or NA cells detected the N protein of RABV with FITC-labeled anti-rabies virus N protein antibodies (Fujirebio Diagnostics, Malvern, USA).

### Construction and Rescue of Mutant RABVs

The RABV vector SAD L16 cDNA clone in pcDNA3.1 (+) was flanked by hammerhead ribozyme and hepatitis delta virus ribozyme sequences as described previously (Schnell et al., 1994; Rasalingam et al., 2005b). The AA residues at K83R and P367S of G in the SAD strain were swapped individually by overlap extension PCR. Briefly, in a PCR reaction, 1 μl of full-length G gene cDNA was mutated and amplified with 20 μM each of 83 or 367 site mutation forward and reverse primers (K83R-F, K83R-R, P367S-F and P367S-R) and 20 μM each of RABV-G forward and reverse primers (RABV-G-F and RABV-G-R) using PrimeSTAR GXL DNA Polymerase (Takara, Dalian, China) according to the manufacturer's instructions (Table 1). The size of PCR products was 1.5 kb, and the products were purified by the GeneJET Gel Extraction Kit. The PCR mixture was heated at 94°C for 2 min, followed by 35 cycles of amplification at 98°C for 10 s, 55°C for 30 s, 68°C for 1 min 45 s, and a final extension at 68°C for 10 min. All G fragments that included AA mutations and the SAD full-length cDNA vector were digested with enzyme set Pst I and BsiW I (Thermo Scientific, Lithuania). The G fragments and SAD full-length cDNA vectors were ligated together in an approximate 1:3 molar ratio using the TaKaRa DNA Ligation Kit LONG (Takara, Dalian, China) according to the manufacturer's instructions.

Recombinant RABVs were rescued as described previously (Faber et al., 2005). Briefly, BSR cells were grown overnight to 80% confluence in 6-well plates in DMEM supplemented with 10% FBS. Cells were transfected with 2.0 μg of the full-length plasmid, 1.0 μg of pH-N, 0.5 μg of pH-P, 0.2 μg of pH-L, and 0.3 μg of pH-G using Lipofectamine™ 3000 Transfection Reagent (Thermo Fisher Scientific, Lithuania) according to the manufacturer's protocol. After incubation for 4 days, the pH of the culture medium was adjusted with 7.5% NaHCO_3_. After incubation for another 3 days, the culture medium was transferred into NA cells and examined for the presence of rescued virus by using FITC-labeled anti-RV N protein antibodies (Fujirebio Diagnostics, Malvern, USA).

### Virus Titration

Viruses were titrated by direct fluorescent antibody assay in NA cells as previously (Zhao et al., 2009). NA cells in 96-well plates were inoculated with serial 10-fold dilutions of virus and incubated at 34°C for 2 days. The culture supernatant was removed, and the cells were fixed with 80% ice-cold acetone for 30 min. The cells were then stained with FITC-labeled anti-RV N protein antibodies. Antigen-positive foci were counted under a fluorescence microscope (Zeiss, Jena, Germany), and viral titers were calculated as fluorescent focus units (FFU) per milliliter. All titrations were carried out in quadruplicate.

### Virus Neutralizing Antibodies (VNA) Assay

VNA titers were measured by the rapid fluorescent focus inhibition test (RFFIT) as previously described (Yang et al., 2015). Briefly, 50 μl of serial 5-fold dilutions of sera were prepared in 96-well plates. A rabies challenge virus (CVS-11) was added to each well and incubated at 37°C for 1 h. Suspended NA cells were added to each well, and the 96-well plates were incubated at 37°C in an incubator with 5% CO_2_ for 24 h. The samples were then fixed with ice-cold 80% acetone for 30 min and stained with FITC-labeled anti-RV N protein antibodies for 1 h at 37°C. After staining, cells were washed three times with PBS, and the results were assessed under a fluorescence microscope. Twenty fields in each chamber were observed, and the 50% endpoint titers were calculated according to the Reed-Muench formula (Fekadu, 1988). The value was compared with the titer of a reference serum obtained from the National Institute for Biological Standards and Control (Herts, United Kingdom).

### Measurement of BBB Permeability

BBB permeability was determined by measuring the uptake of sodium fluorescein (NaF) as described previously. Briefly, 100 μl of 100 mg/ml NaF was injected into each mouse through the tail vein. After 10 min, peripheral blood was collected and the brain was perfused with PBS. The serum of peripheral blood was mixed with an equal volume of 10% trichloroacetic acid (TCA) and was then centrifuged for 10 min. After centrifugation, the supernatant was collected and made up to 300 μl by mixing with 5 M NaOH and 7.5% TCA. The brain was homogenized and centrifuged for 10 min at 10,000 × g, and the precipitate was mixed in cold 7.5% TCA. The supernatant was made up to 300 μl by adding 5 M NaOH. The fluorescence of serum and brain homogenate samples was measured with a spectrophotometer (Varioskan Flash, Thermofisher Scientific) under an excitation spectrum at 485 nm and emission spectrum at 530 nm. The amount of NaF infiltrated into the brain is expressed as the fluorescence micrograms per milligram divided by the fluorescence micrograms per microliter of serum to normalize the amounts of tracer absorbed from the peripheral blood at the time of brain tissue collection. Data was described as the fold change between the quantity of tracer in the tissues from virus-infected mice and the quantity in tissues from the uninfected group.

### Isolation and Culture of bmDCs

Bone marrow–derived dendritic cells (bmDC) are collected and derived from bone marrow of C57BL/6 mice. Mouse bone marrow was removed from the femur bones of C57BL/6 mice. Then, the mouse bone marrow was lysed with lysis buffer (Solarbio, Beijing), and bone marrow stem cells were plated in DC medium [RPMI 1640 medium with 10% FBS, 20 ng/ml granulocyte-macrophage colony-stimulating factor (GM-CSF); PeproTech, USA)] in a 10-cm non-treated Petri dish at 10^6^ cells/ml. Half of the medium was removed on days 3 and 5, and fresh medium containing 20 ng/ml GM-CSF was added. The immature DCs are ready to use on the 7th day postisolation for the DCs and bEnd.3 cells coculture system. After 7 days, the DCs were collected and co-cultured with 80% monolayer bEnd.3 cells in DC medium. After 1 day, the cells were collected with PBS containing 2 μM EDTA.

### MTT Assay

Cell viability was used to determine the suitable SB-3CT (Abcam, USA) concentration of cell treatment. Briefly, cells were seeded on 96-well plates at a density of 5 × 10^4^ cells per well, and the cells were treated with gradient SB-3CT for 24 h to determine cell viability. Next, cells were treated with 0.5 mg/ml MTT (Sigma, UK) at 37°C for 4 h. The mixture discarded and 100 μl DMSO (Sigma, France) added. After mixing for 5 min, the absorption values were measured at 492 nm on a spectrophotometer (Varioskan Flash, Thermofisher Scientific).

### Western Blot

The cells or purified virions were lysed in hot Laemmli sample buffer and boiled for 5 min. Proteins were analyzed on a 10% SDS-PAGE gel and transferred onto a polyvinylidene difluoride (PVDF) membrane. After a 1-h block with 5% bovine serum albumin (BSA) in Tris-buffered saline-Tween, the membrane was stained with anti-RABV-G (G53, 1:2,000) or anti-RABV-N (N42, 1:1,500) overnight. Anti-RABV-G (G53) or N (N42) were prepared as previously described (Jiang et al., 2010). The membranes were then washed with Tris-buffered saline-Tween and incubated for 1 h with horseradish peroxidase-conjugated secondary antibodies (Goat pAb to Rb lgG, ab97051, Abcam, 1:4,000; HRP-conjugated Affinipure Goat Anti-Mouse IgG (H+L), SA00001-1, Proteintech, 1:4,000) at room temperature. All of the blots were developed by the west pico chemiluminescent substrate (Thermo Fisher Scientific, United States). The integrated optical density (IOD) of band signals was analyzed by the Image-Pro Plus 6 (Media Cybernetics, United Kingdom).

### Apoptosis Assay

BSR or NA cells were infected with different RABVs for 48 h at a multiplicity of infection (MOI) of 0.01. Cells were collected at 48 hpi and apoptosis was detected using the FITC Annexin V Apoptosis Detection Kit I (BD biosciences, USA). Briefly, cells were washed twice with cold PBS and then resuspended in 1X binding buffer. Then, cells were added to 5 μl of FITC Annexin V and 5 μl propidium iodide and incubated for 15 min at RT (25°C) in the dark. Flow cytometry analysis was performed on BD AccuriTM C6 flow cytometry (BD Bioscience, USA), and data were analyzed by BD FACSDiva (BD Pharmingen, USA) and FlowJo software (Tree Star, Ashland, OR, USA).

### Statistics Analysis

Statistical analysis of all data is performed by GraphPad Prism software (GraphPad Software, Inc., CA). Virus titer, virus gene copies, and cell infection ratio were evaluated by unpaired two-tailed *t*-test. The survival rate was determined by the log-rank test and Kaplan-Meier survival analysis. For all tests, the following notations were used to indicate significant differences between groups: ^*^P < 0.05; ^**^P < 0.01; ^***^P < 0.001.

## Result

### RABV CNIM1701 Passage in Suckling Mice

**Figure 1:**
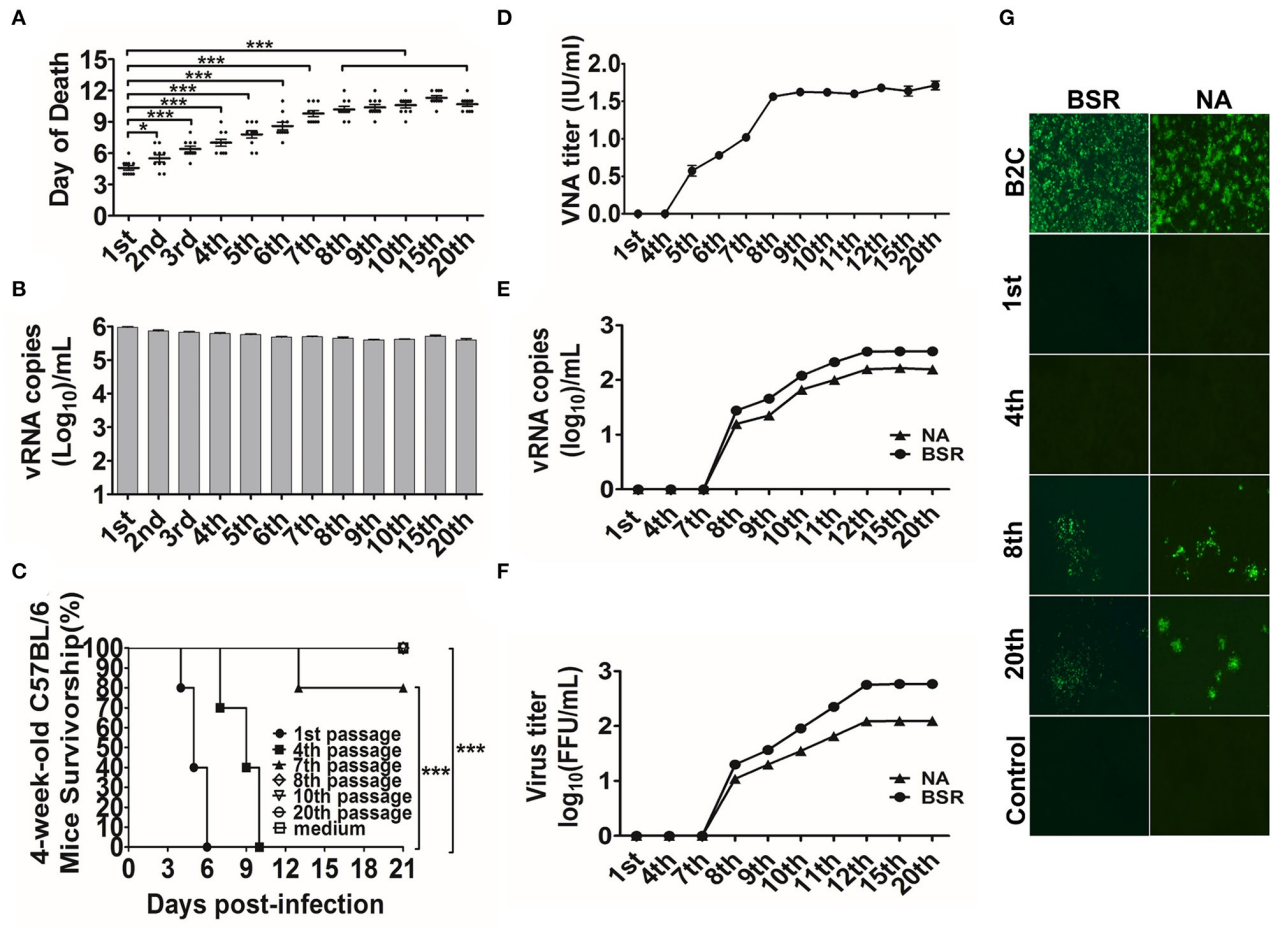
CNIM1701 was adapted to culture cells after 20 passages in the suckling mice. **(A)** Survivorship of 1-day-old C57 BL/6 mice intracranially inoculated with CNIM1701 for 20 passages. Each group contained 10 mice, and all mice were euthanized after rabies clinical sign. **(B)** The genomic RNA of each passage RABV was detected by qRT-PCR. **(C)** Survivorship of 6-week-old C57 BL/6 mice infected with the indicated RABV through IM inoculation. **(D)** VNAs in mice were infected with each passage of RABV. **(E)** The viral genomic RNA of different generations of CNIM1701 was detected in BSR or NA cells by qRT-PCR after 48 h infection. **(F)** The virus titer of different generations of CNIM1701 was detected in BSR or NA cells with FITC-labeled anti-rabies virus N protein antibody after 48 h infection. **(G)** BSR and NA cells were infected with each passage CNIM1701 for 48 h and stained using FITC-labeled anti-rabies virus N protein antibody at 200 × magnification. Statistical analysis of group differences was performed using unpaired *t*-test or log-rank test. A value of *p < 0.05, **p < 0.01, or ***p < 0.001 was considered statistically significant.

To attenuate the wild-type RABV CNIM1701, the CNIM1701 was carried out to 20 serial passages in the suckling mouse brain (Figure 1A). From passages 7 to 20, all sucking mice developed clinical signs 9 days after inoculation, and all passages of RABVs caused a mortality rate of 100% in suckling mice. Due to the faint infection of the original CNIM1701 on NA or BSR cells, the virus titer was difficult to determine, and the gene copy number of RABV was calculated to determine the amount of RABV. The viral genome copy numbers of mouse brain tissue homogenization from passages 1 to 20 were maintained around 10^5.5^ to 10^6^ gene copies/ml (Figure 1B).

To assess the viral pathogenicity in adult mice after continuous passage in suckling mouse brain, 6-week-old C57BL/6 mice were inoculated with different passages of CNIM1701. Briefly, each mouse was inoculated with 10^4^ gene copies of RABV by IM injection. As shown in Figure 1C, the original CNIM1701, the CNIM1701 at the 4th passage, and CVS-11 caused 100% mortality in 6-week-old C57BL/6 mice. After the 8th passage, the CNIM1701 had no mortality in adult mice. Moreover, all the survival mice showed no clinical signs of rabies and had no detected RABV titer in the brain, and the average VNA of survival mice (from the 5th to the 8th passage) rose from 0.57 to 1.68 IU/ml (Figure 1D). The viral propagated properties of each passage of CNIM1701 on culture cells were investigated. As shown in Figures 1E–G, the viral replication of the original and 4th passage CNIM1701 in NA and BSR cells was restricted. After the 8th passage infections in suckling mouse brain, the RABV CNIM1701 was replicated in BSR and NA cells. As expected, the virus genomic RNA copy numbers (from 0 to 10^2.52^ /ml) and virus titer (from 0 to 10^2.8^ FFU/mL) in NA and BSR cells were increased gradually from the 7th to the 20th passage. These results indicate that CNIM1701 after 20 continuous passages in suckling mouse brain was adapted to NA and BSR cells, and this RABV CNIM1701 was renamed CNIM1701-P20.

### Genome Comparison of CNIM1701 and CNIM1701-P20

For comparison of the genetic variation between the different passages of CNIM1701, 10 pairs of primers were designed based on the previously published sequence (Table 1). The complete genome sequence of CNIM1701 was composed of 11,884 nucleotides. The genomic organization was composed of a 58 bp 3′leader RNA, a 1,353 bp of N gene (72 to 1,424), an 894 bp of P gene (1,515 to 2,408), a 609 bp of M gene (2,497 to 3,105), a 1,575 bp of G gene (3,317 to 4,891), a 6,384 bp of L gene (5,410 to 11,793), and a 91 bp 5′trailer.

**Figure 2:**
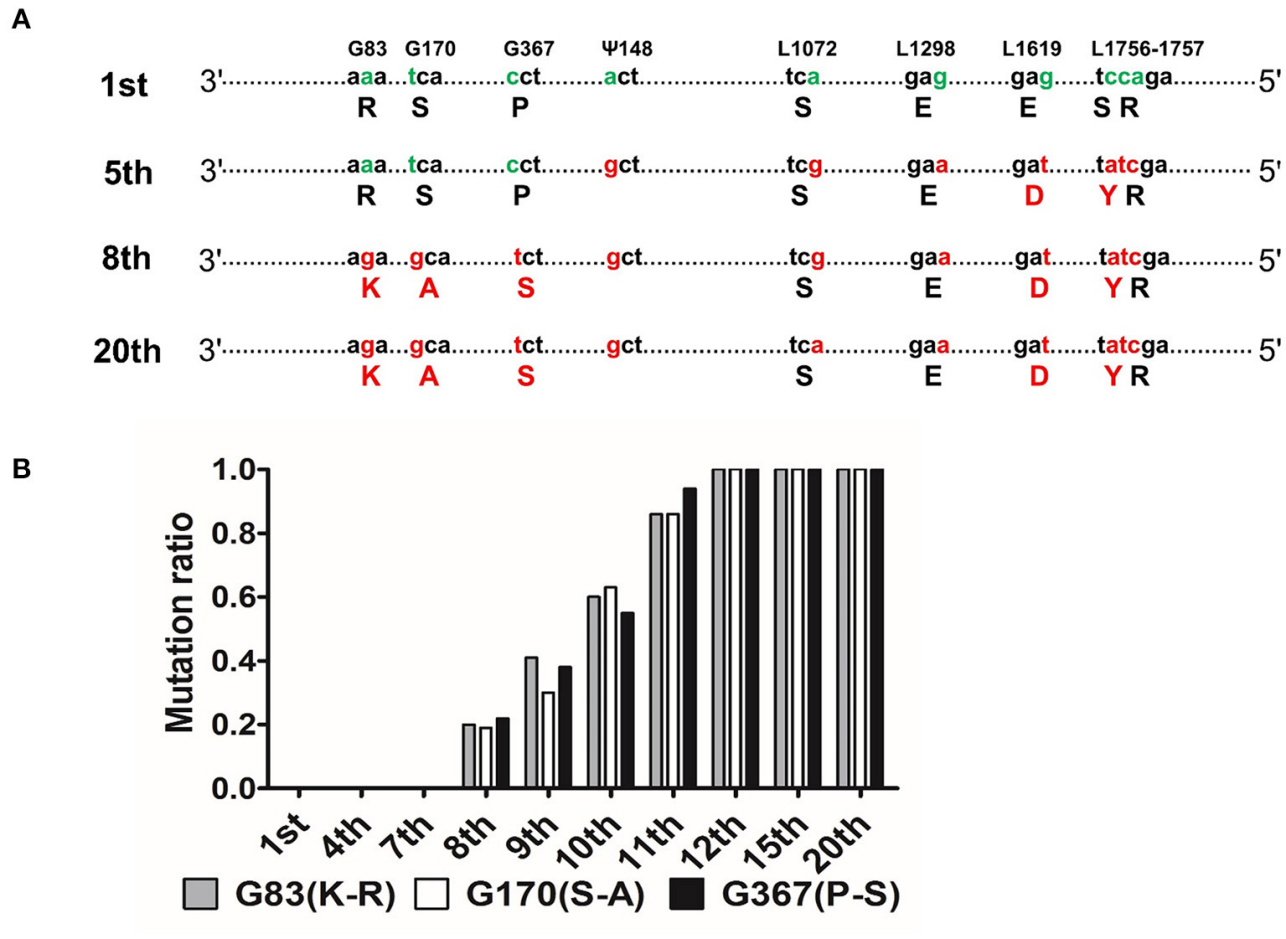
Comparison of genomic nucleotides between the different passages of CNIM1701. **(A)** Schematic diagram of sequence variations between different passages of CNIM1701. Green characters stand for the original AAs and the red characters stand for the mutant AAs that changed after passage in mouse brain. **(B)** Each generation of the whole genome was sequenced 5 times, and the proportion of mutation sites happened in each generation of the virus.

CNIM1701 and the CNIM1701-P20 share 99.7% nucleotide sequence identity, and individual N, P, M, G, and L proteins between the CNIM1701 and the CNIM1701 (15th passage) share an AA sequence homology of 100, 100, 100, 99.8, and 99.9%, respectively. Sequencing analysis revealed 10 nt variations between the original CNIM1701 and CNIM1701-P20 (genomic positions 3,621, 3,881, 4,472, 5,336, 8,663, 9,312, 10,266, 10,676, 10,677, and 10,678), and these mutations resulted in 8 AA substitutions (protein locations G83, G170, G367, Ψ148, L1072, L1298, L1619, and L1756; Figure 2A). All of these mutations happened after 5 passages in suckling mouse brain, and the viral genome maintained stably for further passage. The mutation of Ψ148, L1072, L1298, L1619, 1619, and L1756 occurred at the 8th passage before the virus adapted to NA and BSR cells. Besides this, the mutation of G83, G170, and G367 was found at the 8th passage after the virus adapted to NA and BSR cells. Interestingly, the mutation at G83, G170, and G367 changed gradually from 20% at the 8th passage to 100% at the 12th passage, and each passage of RABV was sequenced 5 times (Figure 2B).

### Phylogenetic Analysis

**Figure 3:**
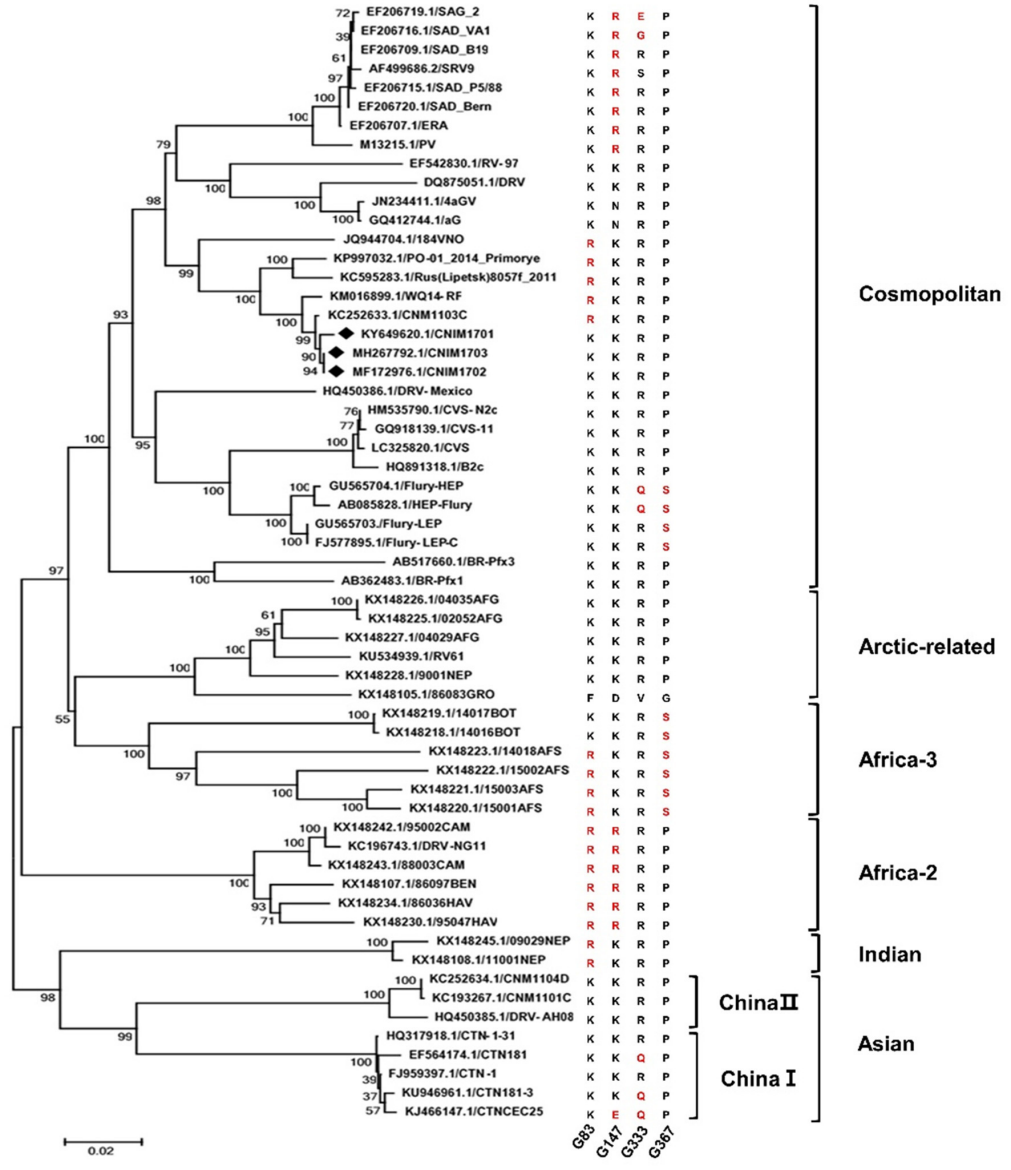
Phylogenetic analysis of the complete genome sequence of CNIM 1701 with other RABV strains. The tree was constructed using the NJ algorithm in MEGA 7.0 software. The numbers below the branches are bootstrap values for 1,000 replicates. CNIM1701, CNIM1702, and CNIM1703 were marked with a black diamond. Alignment of AA sequences of G with all these 59 RABVs. The arginine (R) is shown in red at G83 and G147. The glutamine (Q) and glutamic acid (E) are shown in red at G333. The serine (S) is shown in red at G367.

To understand the phylogenetic relationships of CNIM1701, the entire genomic sequence of these RABVs was aligned with the other 59 genomic genome sequences of RABVs, which are clustered into six major well-defined clades around the world, such as Africa-2, Africa-3, Arctic-related, Asian, cosmopolitan, and Indian clades (Figure 3; Troupin et al., 2016). In the present study, we also isolated 2 other wild-type RABV CNIM1702 (from a rabid bovine) and CNIM1703 (from a rabid sheep). The CNIM1701, CNIM1702, CNIM1703, WQ14-RF, CNM1103C, and other RABV strains that were isolated from Russia or north of China belong to the cosmopolitan clade. In the cosmopolitan clade, 18 of them were RABV vaccine strains, such as PV, CTN, SAD, Flury, CVS-11, aG, and their variants. Others were wild-type RABVs that were directly isolated from animal tissue. These results suggest that the CNIM1701 strain RABVs were more closely related toPV, SAD, Flury, CVS-11, and their variants. Therefore, the historical isolates of original PV, SAD, Flury, and CVS-11 may have the same characteristics at the genomic level. Because the major cell-adapted seed RABV for the vaccine still belonged to three original historical isolates—PV, SAD, and Flury—the original CNIM1701 strain and cell adaptive strain CNIM1701-P20 are an ideal model to examine the initial AA mutations of PV, SAD, and Flury from the original wild-type RABV adapted to cells.

The parental CNIM1701 and CNIM1701-P20 strain RABV have similar genomes but differ in their cellular adaptation. Sequencing analysis revealed that only 3 mutations (G83, G170, and G367) were found to be associated with cellular adaptation properties. To assess whether G83, G170, and G367 occurred in other strains of RABVs, 59 strains of RABVs were determined. Interestingly, CNIM1701-P20, Flury, and Africa-3 have Ser at G367, and CNIM1701, CVS, PV, SAD, and other RABVs have Pro at this position. Furthermore, nearly all RABVs from Northern China have a negligible site in G83 as R, and other RABVs have K at G83. However, the G170 (from S to A) is not related to other RABVs. Our data indicates AA K83R and S367P in RABV glycoprotein may reduce the virulence of RABVs.

### Replacement of AA Residue 83 and 367 in RABV Glycoprotein

**Figure 4:**
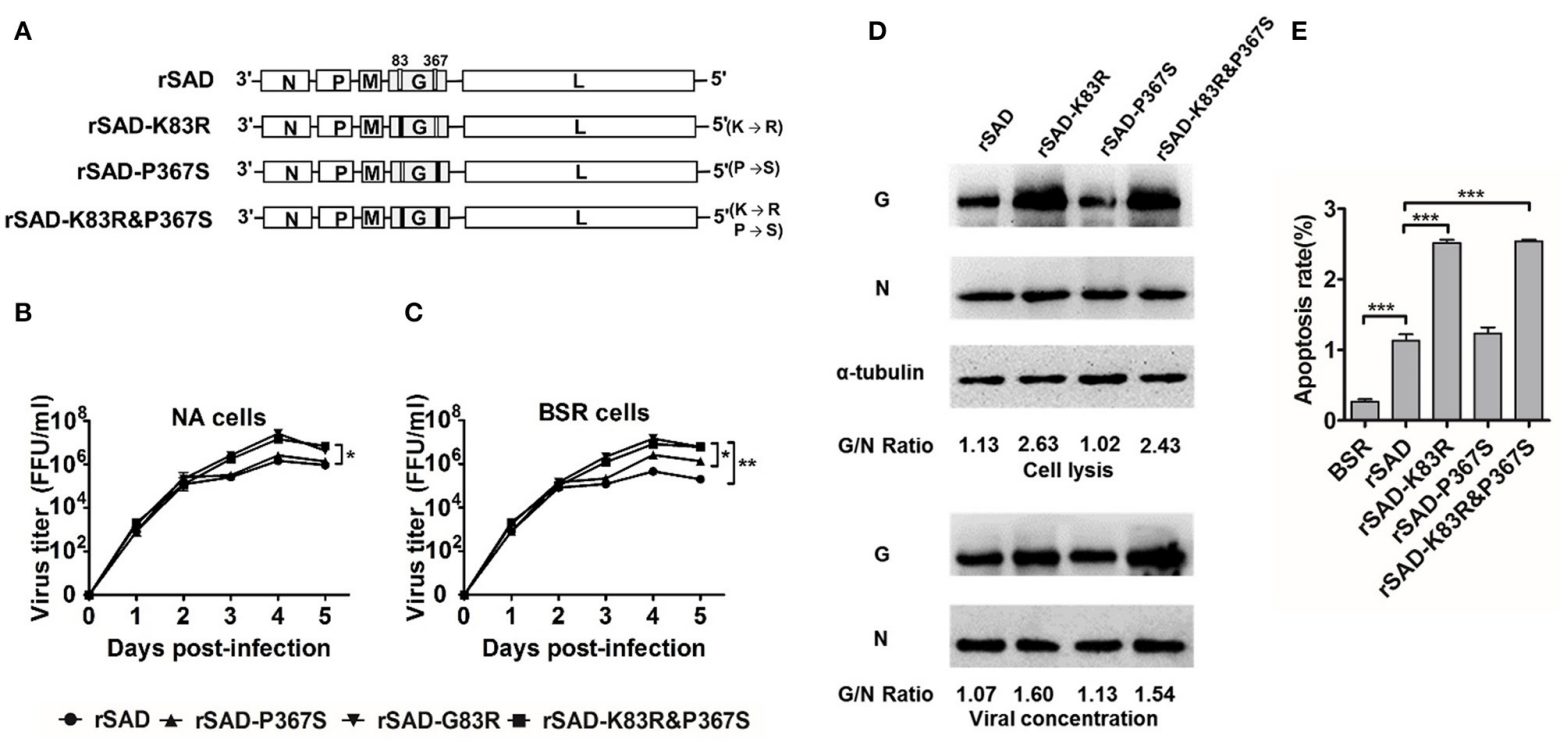
Determination of the replicative rate of rSAD-K83R, rSAD-P367S, and rSAD-K83R&P367S. **(A)** Schematic diagram of the construction of rSAD-K83R, rSAD-P367S, and rSAD-K83R&P367S. **(B,C)** The growth curves of the recombinant RABVs determined on BSR and NA cells, respectively. Briefly, cells were infected with the indicated virus at an MOI of 0.01. The culture supernatants were collected at 1, 2, 3, 4, and 5 dpi and viral titers determined. **(D)** Western blot analysis of viral protein in NA cells and viral protein incorporated into the virion. The ratio between G and N was calculated from the intensity of the band using Image-Pro Plus 6.0. **(E)** Flow cytometry analysis of apoptosis cells infected with the indicated virus. All the titrations were carried out in quadruplicate, and each value was expressed as the mean ± SEM from three independent experiments. Significance of differences between the indicated group was assessed by the unpaired t-test. *p < 0.05; **p < 0.01; ***p < 0.001.

The CNIM1701 strain is a wild-type RABV that produces infection in cells, and it is difficult to reverse genetics to rescue this virus. Furthermore, the SAD strain RABV was in the same clade with CNIM1701 and encoded the same AA at G83 and G367 with CNIM1701 that is different from CNIM1701-P20. To further determine whether the G83 and G367 influence the pathogenicity of RABVs, these AA substitutions were introduced to SAD. Four chimeric recombinant RABVs, rSAD, rSAD-K83R (exchange Lys83 with Arg83), rSAD-P367S (exchange Pro367 with Ser 367), and rSAD-K83R&P367S (exchange Lys83 with Arg83 and Pro367 with Ser 367), were rescued from the genomes of SAD, respectively (Figure 4A). We compared the cellular adaptation of these viruses using the single-step growth curve in NA and BSR cells. Single-step growth curves showed that rSAD-K83R and rSAD-K83R&P367S had similar growth kinetics, whereas the rSAD-P367S has similar growth kinetics as the parental rSAD (Figures 4B,C). At 5 dpi, the virus titer of rSAD-K83R or rSAD-K83R&P367S was 16.3- or 9-fold higher than rSAD in NA cells, respectively. Similarly, the virus titer of rSAD-K83R or rSAD-K83R&P367S was 29- or 20-fold higher than rSAD in BSR cells, respectively.

Generally, the cellular adaptation and immunogenicity of RABVs are related to the G expression (Morimoto et al., 1999; Zhang et al., 2013; Li et al., 2019). To investigate whether these site mutations affect the G expression of these recombinant RABV, we used the Western blot to detect the G protein expression level of infected with indicated RABVs. At 48 hpi, the G expression of rSAD-K83R and rSAD-K83R&P367S was significantly higher than other viruses (Figure 4D). The previous study has demonstrated that the G expression plays an important role in triggering apoptosis, which induced an antiviral immune response and attenuated the RABV pathogenicity (Faber et al., 2002). Therefore, we determined the annexin V expression in the RABV-infected neuronal cells. The proportion of apoptotic cells infected by rSAD-K83R or rSAD-K83R&P367S was 2.51 or 2.54% (Figure 4E). The valves were significantly higher than the cells infected by rSAD (1.13%). These data suggest that G83 change from K to R, which enhances the cellular adaptation of RABVs and G expression, which further support that G83 is essential for virus replication and viral attenuation.

### K83R in Glycoprotein Attenuated RABV and Enhanced the Virus-Induced BBB Permeability

**Figure 5:**
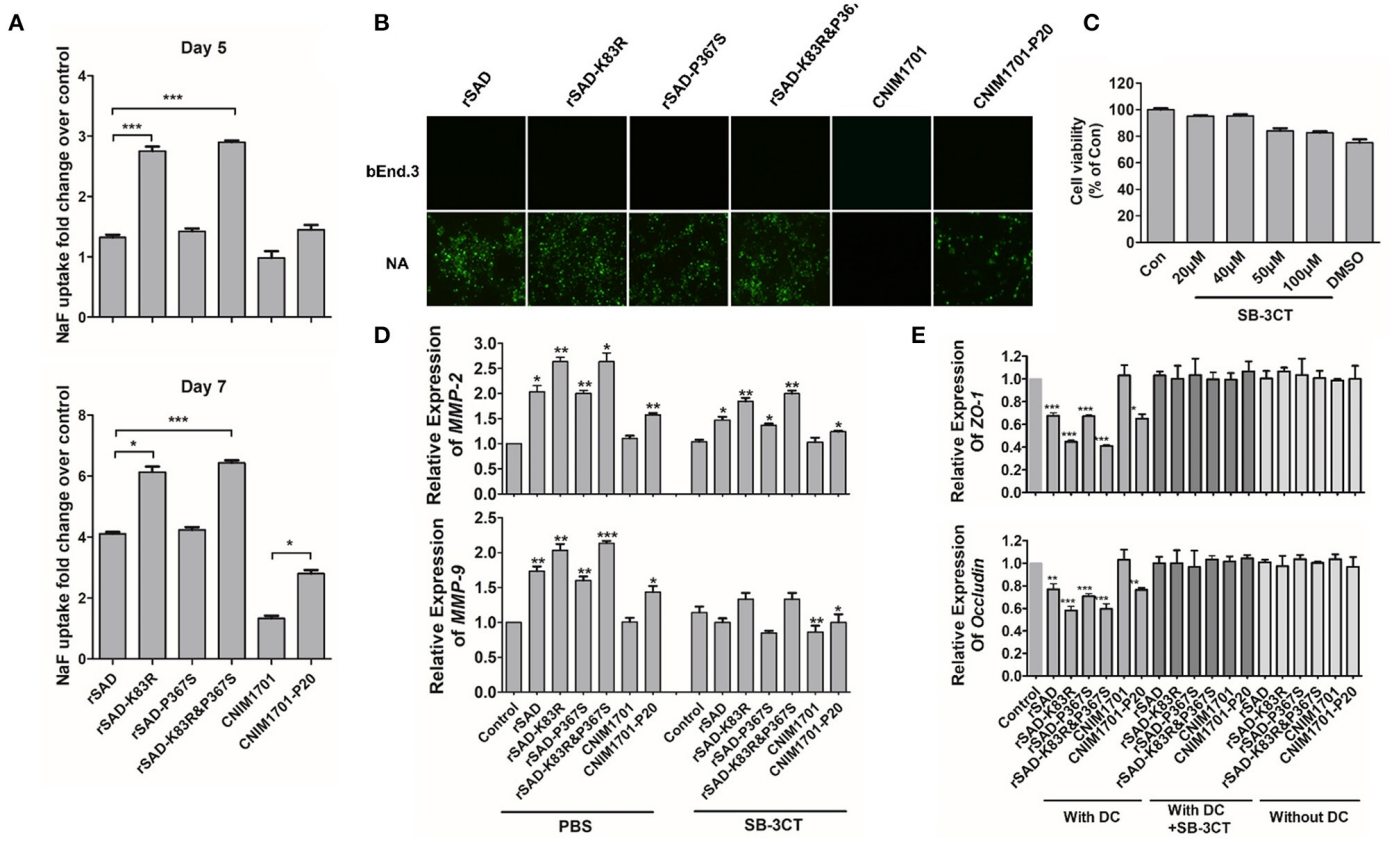
The impact of different viruses on the MMPs and permeability of the BBB. **(A)** C57BL/6 mice were infected with 104 gene copies of RABV by intracranial (i.c.) injection. At day 5 or 7 p.i., the absorption of NaF in the cerebrum was measured. **(B)** The bEnd.3 cells were infected with rSAD, rSAD-K83R, rSAD-P367S, or rSAD-K83R&P367S at MOI of 1, respectively. The fluorescence microscope image (magnification ×200) of cells were stained with FITC-labeled anti-rabies virus N protein antibodies at 48 h after infection. NA cells infected with each virus were included as positive controls. **(C)** Cell viability of DCs after treatment with SB-3CT determined by MTT assay. **(D)** The mRNA level of MMP-2/9 in RABV-infected DCs with or without the treatment of SB-3CT or PBS. **(E)** The mRNA level of ZO-1 and occludin in response to the indicated treatment in bEnd.3 cells. bEnd.3 cells were co-cultured with DCs, RABV-infected DCs, or DCs+SB-3CT. Significance of differences between the indicated group was assessed by the unpaired *t*-test. *p < 0.05; **p < 0.01; ***p < 0.001.

Previous studies demonstrate that laboratory-attenuated RABV but not wild-type RABV enhanced BBB permeability via IM or intracranial infection (Kuang et al., 2009; Chai et al., 2014). To determine whether the attenuated CNIM1701-P20 and K83R mutated RABVs could enhance the BBB permeability, the leakage of NaF from the peripheral circulation to the CNS was measured in the cerebra of RABV-infected mice to investigate the changes of BBB permeability. At 5 days postinfection (dpi), the uptake of NaF in the brains of CNIM1701-P20-infected mice was increased 2-fold compared with those infected with CNIM1701. This is consistent with the results of previous studies (Kuang et al., 2009; Chai et al., 2014). Compared with mice infected with rSAD, the uptake of NaF in the cerebra of mice infected with rSAD-K83R or rSAD-K83R&P367S increased by more than 1.5-fold, but rSAD-P367S hardly changed. This indicates that the BBB was significantly more permeable in the cerebra of mice infected with rSAD-K83R or rSAD-K83R&P367S than in those infected with rSAD. On the 7th dpi, it was observed that the absorption of NaF in the cerebra of all RABV-infected mice increased and BBB permeability was enhanced. The BBB permeability in the cerebra of rSAD-K83R- and rSAD-K83R&P367S-infected mice was higher than rSAD at 7 dpi (Figure 5A).

As shown in Figure 5B, RABVs can replicate effectively in NA cells. However, replication of RABV in bEnd.3 cells was restricted and could not directly enhance the BBB permeability. To determine whether the enhancement of BBB permeability is related to the immune response of RABV, RABV-infected DCs were co-cultured with bEnd.3 cells. One of the key mechanisms of BBB breakdown is damage to the tight junction (TJ) that is composed of both transmembrane (occludin and claudins) and cytosolic TJ proteins (zonula occludens-1 [ZO-1]) (Song et al., 2015). When RABV-infected DCs were co-cultured with bEnd.3 cells, the mRNA levels of ZO-1 and occludin were significantly decreased in bEnd.3 cells. Furthermore, the mRNA levels of ZO-1 and occludin in rSAD-K83R-infected DCs was lower than those of rSAD infection (Figure 5D). Previous studies show that MMP-2/9 may degrade TJ proteins, causing the leakage of the brain barrier (Song et al., 2015). The mRNA level of MMP-2/9 in DCs infected with rSAD-K83R and rSAD-K83R&P367S was significantly higher than that in the uninfected control (Figure 5E). The SB-3CT was used to inhibit the MMP-2/9 expression in the co-cultivation system (Chen et al., 2018). As shown in Figure 5C, 50 μM MSB-3CT inhibited cell viability, so 20 μM SB-3CT was used to inhibit the expression of MMP-2/9. The SB-3CT could effectively decrease the mRNA levels of MMP-2/9 in DCs infected with rSAD-K83R and rSAD-K83R&P367S (Figure 5D) and could also block the downregulation of ZO-1 and occludin induced by RABV-infected DCs (Figure 5E). These data indicate that the change of G83 from K to R enhances the expression of MMP-2/9 in RABV-infected DCs and induces the degradation of TJ protein, which leads to the dysfunction of the BBB-related protein.

### Pathogenicity of Recombinant and Parent RABVs in Mice

**Figure 6:**
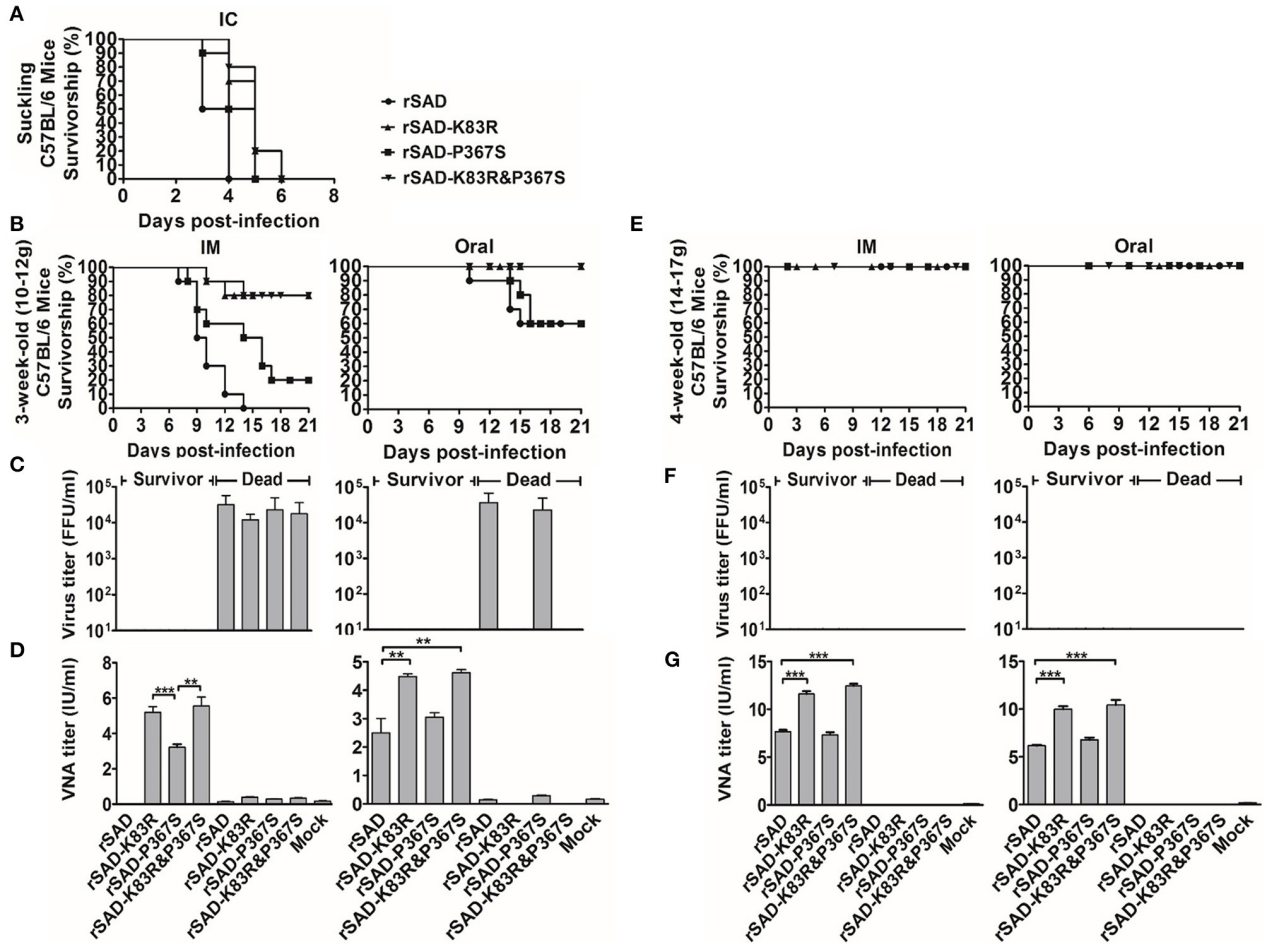
The change of G83 from lysine (K) to arginine (R) in glycoprotein attenuates the pathogenicity of the RABV. **(A)** The survivorship of 1-day-old C57BL/6 mice was inoculated with the indicated virus by IC. **(B,E)** The survivorship of 3- (10–12 g) and 6-week-old (18–22 g) C57BL/6 mice were inoculated with the indicated virus by IM or oral administration. **(C,D)** The virus titer in the brains of dead and surviving mice, which was inoculated with the indicated virus by IM (left) or oral administration (right). **(F,G)** The VNA in the blood of dead and surviving mice, which was inoculated with the indicated virus by IM (left) or oral administration (right). Significance of differences between the indicated group was assessed by the unpaired t-test. *p < 0.05; **p < 0.01; ***p < 0.001.

The effects of K83R and P367S on pathogenicity were assessed *in vivo* with different inoculated routes in suckling and 3- and 6-week-old mice. For evaluation of the pathogenicity in suckling mice that were inoculated by IC with 10^3^ FFU of rSAD, rSAD-K83R, rSAD-P367S, and rSAD-K83R&P367S, all mice died within 7 dpi (Figure 6A). However, the day of death in mice infected with rSAD-K83R was longer than the parental virus. To determine the pathogenicity in adult mice, 3- or 6-week-old mice were inoculated IM or by oral administration with 10^4^ FFU of indicated RABVs, respectively. For 3-week-old mice, inoculating IM with 10^4^ FFU of rSAD-K83R or rSAD-K83R&P367S induced 20% death of mice, and 80% of mice infected with rSAD-P367S succumbed to infection (*P* < 0.05). In contrast, 100% of mice infected with 10^4^ FFU of parental rSAD succumbed to infection (Figure 6B, left). Interestingly, 40% of the mice inoculated by oral administration with 10^4^ FFU of rSAD-P367S or rSAD died, and the rSAD-K83R or rSAD-K83R&P367S was non-lethal to mice by oral administration (Figure 6B, right). In mice that succumbed to rabies, all kinds of RABV titers reached 10^4^ FFU/ml, and the VNA was less than 0.5 IU/ml (Figures 6C,D). In the mice that were infected with rSAD-K83R or rSAD-K83R&P367S, an average VNA production of 5 IU/ml was detected, and RABVs were not detected in the brain of the survivors. For 6-week-old mice, all strains of RABVs were non-pathogenic (Figures 6E–G). As previously reported, the SAD and its derivatives remained pathogenic for young foxes, dogs, and rodents, including mice (Vos et al., 1999; Rasalingam et al., 2005a; Geue et al., 2008). Consistent with previous studies, 40% of adult mice that were orally inoculated with SAD L16 have died 10 dpi. Conversely, the rSAD-K83R was non-lethal to the mice by oral inoculation. These results indicate that the rSAD-K83R was safe for mice with oral immunization.

## Discussion

Rabies is a fatal but preventable infectious disease caused by infection with the RABVs. Lots of rabies vaccines have been developed by attenuating in animals or cell cultures for more than a century (Wu et al., 2011). However, the molecular basis of RABV attenuation is still not fully illuminated. The major cell-adapted seed RABV for the vaccine still belongs to three original historical isolates: PV, SAD, and Flury (Singh et al., 2017). The genetic relationship and molecular differences between the historical isolate and their derivatives are still hard to trace back. Lots of sequence differences are reported between the different SAD strains (Geue et al., 2008), indicating it is hard to define the genome difference between the different pathogenisis of RABV when using different RABV strains. In the present study, we isolated pathogenic RABV from rabid cattle, which was lethal for adult mice and restrictively replicated in the cell cultures. After 20 serial passages on the suckling mice, the CNIM1701-20 was safe for the adult mice and adapted to the cell culture. It would be an ideal model for analysis of the molecular difference between the attenuated and wild-type RABV.

It is reported that the high G protein expression level in attenuated RABV results in strong DC activation and induces a high level of VNA (Faber et al., 2009; Zhang et al., 2013; Li et al., 2019). Other studies show that the G expression level was positively correlated with virus-induced apoptosis and restricted the virus spread speed among neurons (Faber et al., 2002). Previous studies report that the apoptotic cell death can induce inflammation and activate innate and adaptive immunity that may be able to prevent and treat infectious disease and cancer (Restifo, 2000). Moreover, large numbers of apoptotic cells were sufficient to trigger DC maturation and processed intracellular antigens from apoptotic cells (Rovere et al., 1998). Therefore, we thought that the G83 on the RABV glycoprotein would enhance the G expression. Using site-directed mutagenesis to exchange Lys83 with Arg83 and Pro367 with Ser 367 in the G protein of the SAD L16 strain of RABV, we show here that Arg83 on the G protein significantly enhanced the G expression over Lys83 on the G protein, and Arg83 induced strong apoptosis. However, the mutation of G367 on RABV did not significantly change the expression level and induced a similar level of apoptosis compared to the parental virus. Whether the pathogenesis of RABV is determined by the G expression level and virus-induced apoptosis remains to be further investigated. This supports the observation that the wild-type CNIM1701 strain RABV was adapted to the culture cells and non-mortal to adult mice when the Lys83 was exchanged to Arg83.

The SAD L16 is a live-attenuated vaccine virus for oral vaccination in various environmental and epidemiological conditions. Since 1983, there have been more than 233 million vaccine baits with the SAD vaccine virus, and only several incidents have been reported (Head et al., 2019). However, the SAD strain and its variants remain pathogenic for some rodents (Vos et al., 1999). To develop more attenuated vaccine strains, a mutant RABV with substitution at the 194 and 333 AA position of the RABV glycoprotein and a chimeric SAD strain overexpressed additional RABV G by inserting another G in the genome, which significantly reduced the pathogenesis of SAD (Faber et al., 2002, 2005; Takayama-Ito et al., 2006; Tao et al., 2010). Several previous studies also show that attenuated RABVs expressing chemokines or cytokines enhance the immune response (Zhao et al., 2010; Wen et al., 2011).

For high-throughput screening of biomarker differences, algorithms have been widely used in immunology, geology, microbiology, and so on due to its convenience, quickness, and accurate prediction (Peng and Zhao, 2020; Zhao et al., 2020a,b,c,d). Phylogeny tree analysis of the virus demonstrates the changes of AA sites during the attenuation of the rabies virus. In our research, we found those SAD strains and their variants discussed above have Lys on 83 and Pro on 367, indicating the SAD strain could be further attenuated with these AA site mutations. Because exchange Lys83 with Arg83 and Pro367 with Ser 367 in the G protein of SAD L16 further attenuated the virus, we can infer that there would be a potential proliferation pathway that optimizes G to enhance immunogenicity.

MMPs participate in many physiological and pathological processes in the brain and BBB (Feng et al., 2011). Previous research has shown that MMPs degrade TJ proteins and basement membrane proteins. Many hosts and viral factors contribute to the disruption of BBB integrity, such as HIV, which alternates the expression of occludin and ZO-1 with viral gp120 and CCL2 (Dallasta et al., 1999). MAV-1 infects ECs and directly affects TJ proteins (Gralinski et al., 2009). Previous studies demonstrate that a lab-attenuated RABV could indirectly affect mouse BBB permeability through infection. Furthermore, the lab-attnuated RABV-infected brain extracts enhanced BBB permeability (Chai et al., 2014). Our results also demonstrate that RABV could not directly affect BBB permeability. However, the lab-attenuated RABV activates the DCs and enhances MMPs, which may indirectly enhance mouse BBB permeability by reducing TJ protein expression and driving the neutralized antibody into the brain. Otherwise, wild-type RABV evades peripheral immune cell recognition, and there may not be accumulated peripheral immune cells that touch the BBB and affect the permeability of the BBB. Another team demonstrates that phosphoprotein of wild-type RABV restricts BBB permeability, which may enhance the pathogenicity of RABV (Long et al., 2020). Combined with our experimental results, which demonstrate that the wild-type RABV hijacked different mechanisms to evade the host immune system. All of this research illustrates that opening the BBB is a key factor for the prevention of RABV infection.

In summary, our studies demonstrate that exchanging the Lys83 with Arg83 in the G protein of the pathogenic RABV can result in reversion to the non-pathogenic phenotype. Furthermore, we used the RABV SAD strain as a model that confirmed that K83R of the G mutation is critical in attenuation of SAD RABVs. Additionally, studies with the chimeric virus revealed that the K83R mutation enhanced the expression level of G protein and BBB permeability.

## Data Availability Statement

The datasets presented in this study can be found in online repositories. The names of the repository/repositories and accession number(s) can be found in the article/supplementary material.

## Ethics Statement

The animal study was reviewed and approved by C57BL/6 mice were purchased from Charles River (Beijing Vital River Laboratory Animal Technology Co., Ltd.). Experimental infectious studies were performed in strict accordance with the Guide for the Care and Use of Laboratory Animal Monitoring Committee of Hubei Province, China, and the Scientific Ethics Committee approved the protocol of Huazhong Agricultural University (protocol no. Hzaumo-2015-018). All efforts were made to minimize the suffering of the animals.

## Author Contributions

YY conceived the study and wrote the manuscript. CL, YW, HL, XZ, DB, and SZ performed all experiments. WH and YY edited the study. All critically reviewed and approved the final manuscript.

## Conflict of Interest

The authors declare that the research was conducted in the absence of any commercial or financial relationships that could be construed as a potential conflict of interest.
